# Sagittal alignment at 3 years old determines future thoracolumbar kyphosis in achondroplasia: A prospective study with minimum 5-year follow-up from infancy

**DOI:** 10.1016/j.xnsj.2021.100070

**Published:** 2021-05-13

**Authors:** Kei Ando, Kazuyoshi Kobayashi, Hiroaki Nakashima, Masaaki Machino, Sadayuki Ito, Shunsuke Kanbara, Taro Inoue, Naoki Segi, Hiroyuki Koshimizu, Shiro Imagama

**Affiliations:** Department of Orthopaedic Surgery, Nagoya University Graduate School of Medicine 65, Tsurumai, Showa-ku, Nagoya, Aichi 466-8550, Japan

**Keywords:** Sagittal alignment, Achondroplasia, Thoracolumbar kyphosis, Natural progression

## Abstract

**Background:**

Little is known about the progression of Thoracolumbar kyphosis (TLK) in achondroplasia. The aim of the study was to evaluate natural progression of TLK and establish risk factors for progression.

**Methods:**

We reviewed 21 patients (11 males, 10 females) diagnosed clinically and radiographically with achondroplasia as infants and followed for a minimum of 5 years from infancy, and analyzed to compare differences between data at 0, 1, 3, 5, 7-10, and 11-18 years old. Subjects (n=21) were divided into two groups with and without TLK >20° at the thoracolumbar junction on lateral standing radiographs at age 3.

**Results:**

TLK >20° occurs in 76.2% of infants in the first 7 months of life. Sagittal parameters at 0, 1, 3, 5, 7-10, and 11-18 years old differed significantly for cervical lordosis (CL), thoracic kyphosis (TK), TLK, lumbar lordosis (LL), pelvic tilt (PT), and sacral slope (SS). TK, LL, and SS increased significantly with increasing age, whereas CL, TLK, and PT were significantly lower in older age groups (*P* < 0.05). In 6 of 7 patients with TLK >20° at age 3, TLK had progressed or was still >20° at age 5. The prevalence of TLK >20° at age 3 was 33.3% (7/21). There was a significant difference in age at independent walking among subjects with and without TLK at age 3 (31.4±17.1 vs. 16.1±3.4 months, *P* < 0.01). Radiologic parameters associated with TLK showed significant differences between subjects with and without TLK at age 3, including TLK, TK, TLK, LL, and SVA at age 5; and TLK at ages 7-10 and 11-18.

**Conclusions:**

These results suggest that sagittal alignment at 3 years old determines future TLK in achondroplasia. Progression of deformity and neurological impairments require consideration in treatment planning.

## Background

Achondroplasia is the most common form of human dwarfism, and the causative mutation may be the most common disease-causing mutation to arise de novo in humans [1,2]. The clearly recognizable features of achondroplasia are shortening of the arms and limbs. The skull exhibits characteristic macrocephaly with frontal bossing, saddlenose deformity, and a narrow foramen magnum and short clivus. There are several characteristics of an achondroplastic spine that make the entire spinal canal narrow and increase the chance of compression of the spinal cord or cauda equina. The pedicles are short, particularly in the thoracolumbar region, and there is reduction of interpediculate spacing of the lumbar vertebrae, which becomes progressively smaller in the caudal direction [3]. Within the spinal column, the thoracolumbar spine is the most frequently affected region, with thoracolumbar stenosis and sagittal deformity being the most common abnormalities [4,5].

A transient thoracolumbar kyphosis (TLK) of 15-25° is present in 95% of infants in the first 6 months of life [6]. With acquisition of a sitting posture between age 6 to 18 months, kyphosis may increase to 60°–70° and often includes T-10 through L-4. As the child begins to ambulate, the rate of kyphosis decreases to 11% by age 10 [6]. However, 10–15% of adults have a fixed, angular kyphosis with marked secondary deformity of one or more vertebrae [6]. Such wedging results in a high risk for significant neurologic consequences, and about half of affected adolescents and adults have problems such as weakness, paralysis, and bladder or bowel incontinence [6], [7], [8]. These problems arise because of draping or tethering of the cord, so that with continued growth the normal “ascent” of the cord is prevented because it is fixed at two sites (the medulla and kyphotic apex) and stretching results in damage to the cord [9].

There are few data on the natural history of TLK in achondroplasia. Establishment of a suitable time and method of intervention for TLK might be possible if factors related to progression of TLK in achondroplasia could be obtained in a longitudinal study. Therefore, the aim of this study was to follow infants for a minimum of 5 years to examine TLK progression and identify factors related to progression.

## Materials and methods

### Patient population

The subjects were 21 patients (11 males,10 females) who were clinically and radiographically diagnosed with achondroplasia in infancy and followed for a minimum of 5 years from infancy. The study was performed from 2002 to 2014. The Institutional Review Board of our institute approved the study and the parents of each patient provided informed consent before enrollment. Six cases were excluded from the study because of a late start (>1 year of age) or insufficient radiographs. All patients were examined at 3- to 12-month intervals in visits to our hospital. The mean age at the first examination was 6 months (range: 1–8 months) and the average follow-up was 92 months (range: 60–192 months) (Table 1).

**Table 1:** Summary of demographic data in 21 subjects.

Sex	
male	11
female	10
Age (years) (initial visit)	0.5 ± 0.3
Age (years) (final follow-up)	8.2 ± 3.5
Hydrocephalus	5 (23.8)
Growth hormone	19 (90.5)
Ventriculoperitoneal shunt	1 (4.8)
Foramen magnum decompression	3 (14.3)
Brace treatment	4 (19.0)
Age (months) of independent walking	21.2 ± 12.2

Data are shown as mean ± standard deviation or number (percentage) of patients

### Clinical parameters

Growth hormone therapy, hydrocephalus, ventriculoperitoneal (VP) shunt, foramen magnum decompression (FMD), and age of independent walking in a family questionnaire were determined as clinical parameters. These parameters were analyzed using a binary scale, indicating the presence or absence of each parameter in subjects with and without TLK.

### Radiologic parameters

A standard protocol was used for all radiographs. Initial radiographs at 1-7 months (0 years old) were taken in a prone lateral position. At later visits, radiographs were taken in sitting and standing positions. All lateral radiographs included both hip joints and the C7 vertebra. The following spinal parameters were assessed: C2-C7 lordosis angle (cervical lordosis; CL), measured as the angulation of intersection between lines parallel to the C2 superior endplate and C7 inferior endplate; T2-T12 angle (thoracic kyphosis; TK), measured as the angulation of intersection between lines parallel to the T2 superior endplate and T12 inferior endplate; thoracolumbar kyphosis (TLK), measured between the upper endplate of T10 and lower endplate of L2; lumbar lordosis (LL), measured as the angulation of intersection between lines parallel to the superior endplate of T12 and the sacrum; pelvic incidence (PI), measured as the angulation of intersection between lines parallel to a line connecting the midpoint of the superior endplate of the sacrum to the center of the hip axis and a perpendicular line to the superior endplate of the sacrum; pelvic tilt (PT), measured as the angulation of intersection between lines parallel to a line connecting the midpoint of the superior endplate of the sacrum to the center of the hip axis and a vertical line; sacral slope (SS), measured as the angulation of intersection between lines parallel to the superior endplates of the sacrum and a horizontal line; and the sagittal vertical axis (SVA), measured as the distance between the sagittal offset of a plumb line dropped from the C7 vertebral body and the posterosuperior corner of the sacral plate. All measurements were made twice by two spine surgeons independently. The apical vertebral wedging angle was measured between the upper and lower endplate of the vertebra if TLK was concomitant with vertebral wedge.

### Statistical analysis

Data were analyzed using Statistical Program for the Social Sciences ver.26 (SPSS Inc., Chicago, IL). Data are shown as mean ± SD. A Friedman test (nonparametric) with one-way repeated measures was used to compare differences between data at 0, 1, 3, 5, 7-10, and 11-18 years old. A Wilcoxon signed-rank test was used to analyze changes between two time points. Significance was set as P < 0.05. Subjects (n=21) were divided into two groups with and without TLK >20° at the thoracolumbar junction on lateral standing radiographs at age 3 [10]. Clinical parameters in the two groups were compared by Pearson chi-square statistic with a Fisher exact test to compensate for the small sample size in one group.

## Results

Sex, mean ages at initial visit and final follow-up, presence of hydrocephalus, growth hormone therapy, VP shunt, FMD, and age of independent walking are shown in Table 1.

Sagittal parameters at ages 0, 1, 3, 5, 7-10, and 11-18 are shown in Table 2. TLK >20° occurs in 76.2% of infants in the first 7 months of life. With acquisition of a sitting posture between 12 and 24 months, kyphosis is postulated to increase to 85.7%, but the rate decreases to 33.3% by age 5. These age groups had significant differences in CL, TK, TLK, LL, PT, and SS. With increasing age, TK, LL and SS were significantly higher, whereas CL, TLK and PT were significantly lower (*P* < 0.05). There was no significant intergroup difference for PI or SVA (*P* > 0.05).

**Table 2:** Sagittal parameters at ages 0, 1, 3, 5, 7-10, and 11-18 years.

							Friedman	Wilcoxon P
	0 y (n=21)	1y (n=21)	3 y (n=21)	5 y (n=21)	7-10 y (n=15)	11-18 y (n=6)	P value	Significance
CL: C2-7 lordosis	9.9 ± 6.5	6.9 ± 4.9	6.5 ± 5.0	7.3 ± 7.5	6.3 ± 9.0	7.9 ± 6.3	<0.05	CL0 vs. CL1 and CL3
TK: Thoracic kyphosis	7.9 ± 5.1	9.6 ± 6.7	22.5 ± 12.8	26.7 ± 12.0	22.5 ± 11.4	23.9 ± 4.1	<0.01	TK0 vs. TK3 and TK5
TLK: Thoracolumbar kyphosis	29.2 ± 12.6	29.2 ± 8.2	18.8 ± 18.5	16.5 ± 18.0	23.4 ± 26.4	26.1 ± 33.1	<0.05	TLK1 vs TLK5.
LL: Lumbar lordosis	24.9 ± 9.5	34.9 ± 18.7	47.8 ± 17.6	53.1 ± 15.1	52.6 ± 14.8	52.4 ± 17.2	<0.01	LL0 vs. LL1,3,5
PI: Pelvic incidence		47.7 ± 14.4	50.5 ± 11.1	51.1 ± 13.0	55.1 ± 14.1	58.0 ± 16.7	ns	
PT: Pelvic tilt		15.3 ± 9.5	9.3 ± 6.8	10.0 ± 7.4	12.5 ± 8.6	17.7 ± 9.9	<0.01	PT1 vs. PT3
SS: Sacral Slope		32.5 ± 9.2	41.2 ± 9.4	41.1 ± 11.2	42.6 ± 10.0	40.3 ± 9.0	<0.01	SS1 vs. SS3 and SS5
SVA (C7S1): Sagittal vertical axis		14.6 ± 46.3	−13.1 ± 33.0	−16.4 ± 35.1	−5.6 ± 44.6	16.8 ± 42.1	ns	
TLK>20 degree	16 (76.2)	18 (85.7)	7 (33.3)	6 (28.6)	5 (35.7)	2 (33.3)		
Apical vertebral wedge	4 (19.0)	7 (33.3)	9 (42.9)	9 (42.9)	8 (53.3)	3 (50)		
Apical vertebral wedging angle	13.3 ± 8.5	15.2 ± 5.2	16.2 ± 6.2	18.4 ± 9.7	18.6 ± 11.1	24.9 ± 20.0		

Data are shown as mean  ±  standard deviation or number (percentage) of patients

ns; not significant

**Fig. 1:**
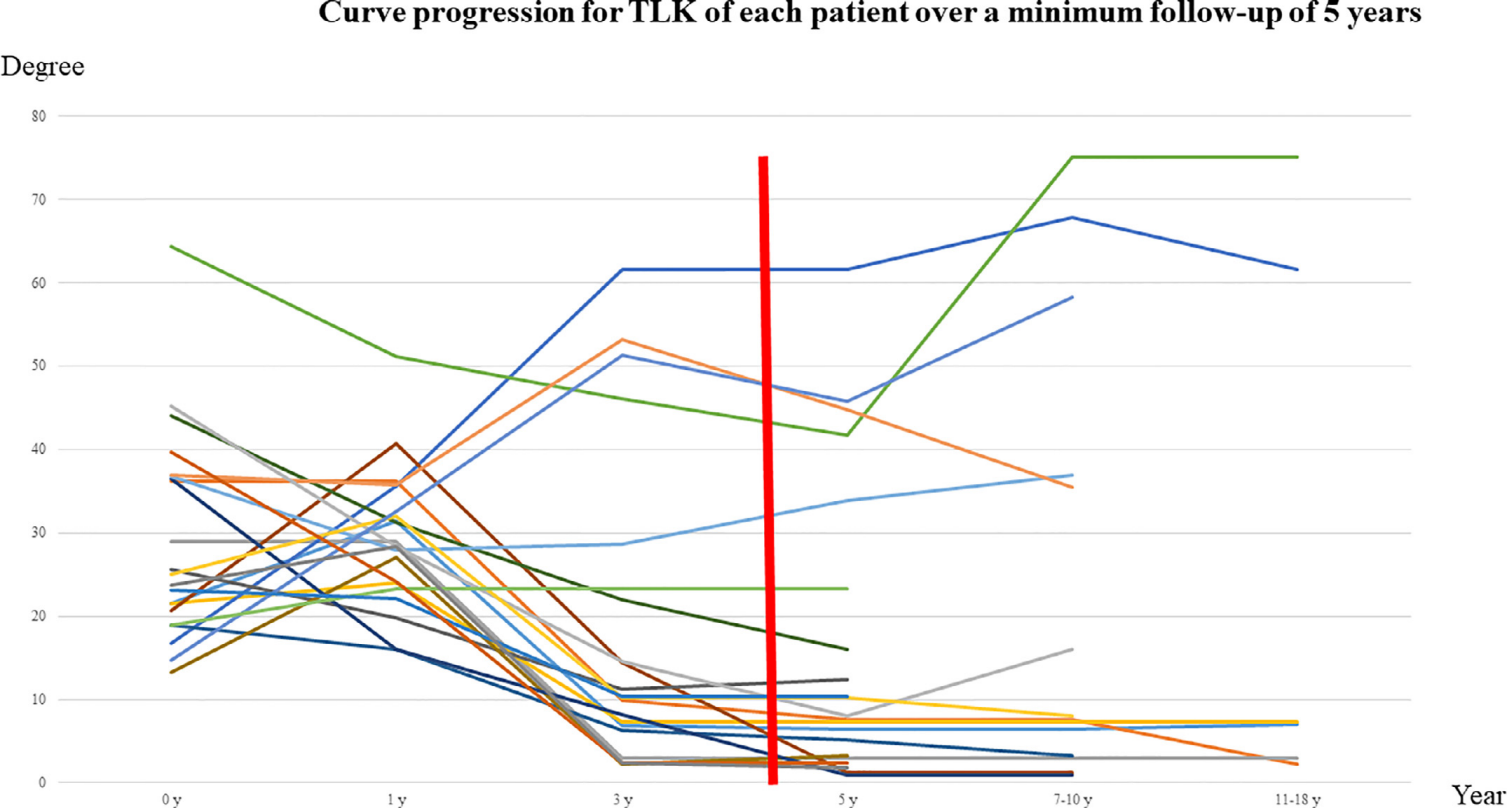
Curve progression for TLK of each patient. Most patients aged 0 to 1 year had TLK >20°. However, 6 of 7 patients with TLK >20° at age 3 years had an increase of TLK or TLK remained at >20° at age 5. All except one of these patients had further progression of TLK or TLK remained at >20° at age 7. Each line is a distinct patient. Vertical red line defined as 3 year.

Curve progression in TLK for each patient is shown in Fig. 1. Most patients aged 0 to 1 had TLK >20°. Six of 7 patients with TLK >20° at age 3 progressed or had TLK >20° at age 5, and all but one of these patients had and had TLK >20° at age 7 (Fig. 1).

The prevalence of TLK >20° at age 3 was 33.3% (7/21). Age at independent walking was significantly higher for those with TLK >20° at age 3 compared to those with a lower TLK (31.4±17.1, vs. 16.1±3.4, *P* < 0.01). There were no significant differences in hydrocephalus, VP shunt, and FMD between the two groups. Radiologic parameters associated with TLK showed significant differences between subjects with and without TLK >20° at age 3, including TLK, TK, TLK, LL, and SVA at age 5; and TLK at ages 7-10 and 11-18 (Table 3).

**Table 3:** Sagittal parameters of achondroplasia in subjects divided into two groups with or without TLK >20° at 3 years old.

	0 y (n=7) with TLK at 3 y	0 y (n=14) without TLK at 3 y	P value	1 y (n=7) with TLK at 3 y	1 y (n=14) without TLK at 3 y	P value	3 y (n=7) with TLK at 3 y	3 y (n=14) without TLK at 3 y	P value
**Age (months) of independent walking**	**31.4 ± 17.1**	**16.1 ± 3.4**	**<0.01**						
CL: C2-7 lordosis	11.6 ± 6.6	9.0 ± 6.6	ns	6.1 ± 4.0	7.2 ± 5.1	ns	6.0 ± 6.2	6.8 ± 4.5	ns
TK: Thoracic kyphosis	11.4 ± 5.6	6.2 ± 4.1	ns	12.1 ± 8.3	8.4 ± 5.7	ns	18.9 ± 12.4	24.2 ± 13.1	ns
TLK: Thoracolumbar kyphosis	33.2 ± 17.9	27.1 ± 9.0	ns	33.9 ± 8.8	26.8 ± 7.1	ns	**40.9 ± 16.0**	**29.2 ± 12.6**	**<0.01**
LL: Lumbar lordosis	21.1 ± 7.4	26.8 ± 10.1	ns	40.3 ± 25.7	32.1 ± 14.4	ns	44.8 ± 26.1	49.3 ± 12.4	ns
PI: Pelvic incidence				47.8 ± 16.3	47.7 ± 14.0	ns	46.1 ± 10.2	52.7 ± 11.3	ns
PT: Pelvic tilt				16.6 ± 9.5	14.6 ± 9.8	ns	8.9 ± 4.9	9.5 ± 7.8	ns
SS: Sacral Slope				31.3 ± 7.6	33.1 ± 10.2	ns	37.2 ± 9.8	48.2 ± 8.8	ns
SVA (C7S1): Sagittal vertical axis							8.5 ± 39.2	−22.1 ± 27.0	ns
Apical vertebral wedge	1 (14.3)	3 (21.4)	ns	5 (71.4)	4 (28.6)	ns	5 (71.4)	4 (28.6)	ns
Apical vertebral wedging angle	16.7	12.2 ± 10.0	ns	17.4 ± 7.1	14.7 ± 5.6	ns	17.4 ± 7.1	14.7 ± 5.6	ns
Growth hormone	7 (100)	12 (85.7)	ns						
Hypocephalus	3 (42.9)	2 (14.3)	ns						
Foramen magnum decompression	2 (28.6)	1 (7.1)	ns						
Brace treatment	3 (42.9)	1 (7.1)	ns						
5 y (n=7) with TLK at 3 y	5 y (n=14) without TLK at 3 y	P value	7-10 y (n=5) with TLK at 3 y	7-10 y (n=9) without TLK at 3 y	P value	11–18 y (n=2) with TLK at 3 y	11–18 y (n=4) without TLK at 3 y	P value	
7.3 ± 7.5	9.9 ± 6.5	ns	6.2 ± 10.7	6.3 ± 8.7	ns	12.3 ± 1.1	5.7 ± 6.8	ns	
**18.5 ± 12.1**	**30.8 ± 10.1**	**<0.05**	15.0 ± 11.8	26.6 ± 9.3	ns	26.7 ± 5.6	22.5 ± 3.2	ns	
**38.2 ± 15.3**	**5.7 ± 3.7**	**<0.01**	**54.7 ± 17.9**	**6.0 ± 4.7**	**<0.01**	**68.4 ± 9.5**	**4.9 ± 2.6**	**<0.01**	
**43.8 ± 19.3**	**57.8 ± 10.5**	**<0.05**	46.7 ± 22.6	55.8 ± 8.3	ns	64.9 ± 14.4	46.1 ± 16.3	ns	
43.8 ± 13.1	54.7 ± 11.8	ns	50.4 ± 16.4	57.7 ± 12.8	ns	62.2 ± 20.7	55.9 ± 17.5	ns	
8.4 ± 6.0	10.8 ± 8.1	ns	13.3 ± 10.0	12.0 ± 8.3	ns	23.4 ± 7.1	14.8 ± 10.7	ns	
35.4 ± 14.5	43.9 ± 8.3	ns	37.1 ± 11.0	45.7 ± 8.5	ns	38.8 ± 13.6	41.1 ± 8.4	ns	
**4.5 ± 36.6**	**-27.6 ± 30.0**	**<0.05**	5.2 ± 53.2	-14.6 ± 38.8	ns	36.0 ± 33.9	7.2 ± 47.0	ns	
5 (71.4)	4 (28.6)	ns	5 (100)	3 (33.3)	ns	2 (100)	1 (25)	ns	
22.3 ± 9.9	13.5 ± 8.1	ns	23.1 ± 11.0	12.6 ± 9.7	ns	34.5	10.7	ns	

Data are shown as mean  ±  standard deviation or number (percentage) of patients.

ns; not significant.

### Illustrative cases

**Fig. 2:**
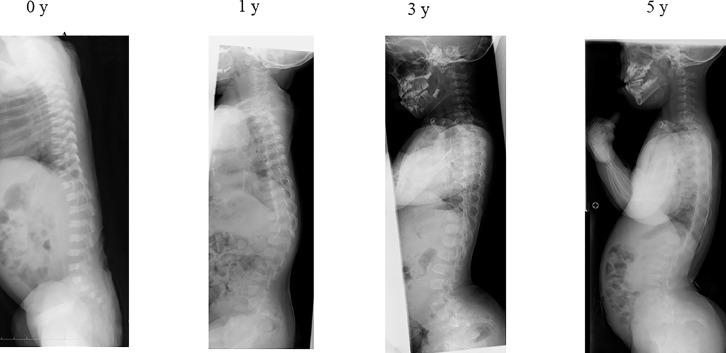
Spontaneously recovered case of TLK. TLK was 15.4° at 0 y and 27° at 1 y, but spontaneously recovered to 2.2° at 3 y and remained low at 3.3° at 5 y. The subject first walked independently at age 24 months.

Case 1: TLK was 15.4° at age 0 and 27° at age 1, but spontaneously recovered to 2.2° at age 3 and 3.3° at age 5 (Fig. 2). Independent walking occurred at 24 months.

**Fig. 3:**
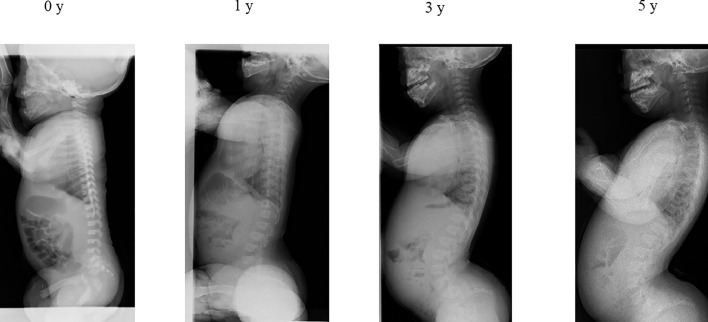
Spontaneously recovered case of TLK with wedging vertebra. TLK was 23.8° at 0 y and 28.4° at 1 y with L2 wedging of 15.7°, but spontaneously recovered to 2.5° at 3 y and remained low at 1.9° at 5 y. The subject first walked independently at age 13 months.

Case 2: TLK was 23.8° at age 0 and 28.4° at age 1 with L2 wedging of 15.7°, but spontaneously recovered to 2.5° at age 3 and 1.9° at age 5 (Fig. 3). Independent walking occurred at 13 months.

**Fig. 4:**
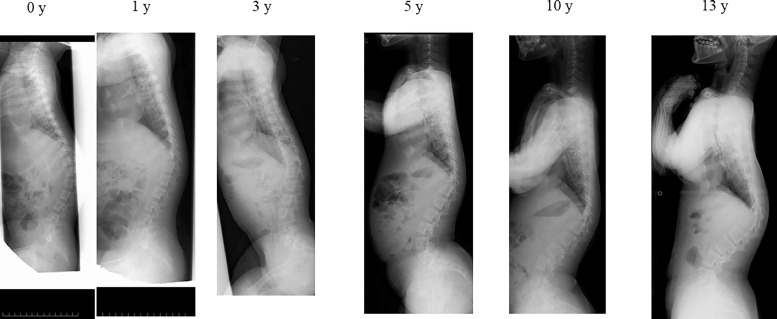
TLK progressed case. TLK was 64.3° at 0 y and 51.1° at 1 y with L1 wedging of 8°, and then progressed to 46° at 3 y, even with brace treatment, and to 75.1° at 10 y. The subject first walked independently at age 48 months.

Case 3: TLK was 64.3° at age 0 and 51.1° at age 1 with L1 wedging of 8°, and remained at 46° at age 3, even with brace treatment, and progressed to 75.1° at age 10 (Fig. 4). Independent walking did not occur until 48 months.

## Discussion

Achondroplasia is the most common form of skeletal dysplasia, with an incidence as high as 1 per 15,000 births [11,12]. A point mutation in the fibroblast growth factor receptor-3 (FGR-3) gene leads to a defect in enchondral ossification, which results in rhizomelic limb-shortening and spinal column abnormalities [2,11,13]. Intracartilaginous ossification commences in the developing fetus in the thoracolumbar region and progresses in a cranial and caudal direction from the thoracolumbar junction. The primary ossification centers are located in the vertebral centrum and one on each side in the posterior elements, located anterior to the pedicle. The junction of these centers is the neurocentral synchondrosis. During maturation, there is increasing vertebral size and progressive expansion of the spinal canal. Fusion of these synchondroses at 6–8 years of age signals cessation of spinal canal widening. This is also the period in which longitudinal growth of the posterior elements of the vertebrae ceases [14]. Anterior longitudinal growth, which occurs at the epiphyseal plates, continues in individuals until age 18–20 years. Therefore, factors interfering with anterior longitudinal growth during the intervening period are accompanied by kyphosis,[8] and most cases have kyphosis at the thoracolumbar junction. If kyphosis progresses there is wedging of vertebral bodies at the apex of the deformity, giving bullet-shaped/hypoplastic vertebrae [15].

At birth, there is normal prominence of the mid-to-lower back with a small TLK, which usually resolves by the time of independent walking [6,14]. Lumbar lordosis then evolves and increases until skeletal maturity. In this study, TLK >20° was present in 76.2% of infants within the first 7 months of life. With acquisition of a sitting posture between 12 and 24 months, kyphosis increases to 85.7%, but then the rate decreases to 33.3% by age 5 years. In contrast to the decrease of TLK, lumbar lordosis and sacral slope increase with aging. In achondroplasia the exact factors associated with progression of TLK and subsequent development of fixed kyphosis in adolescence or adulthood are not fully identified, but are thought to be connected to generalized hypotonia, a large head, hydrocephalus, and delayed walking [6,7,10]. Siebens et al. presented a hypothesis explaining the persistent curvature and resultant TLK in achondroplasia, in which deformity was related to a disproportionately enlarged head and lax ligaments with an unusual vertical load on the anterior column of the spine. This is most apparent at the thoracolumbar junction where the transition from the relative rigidity of the thoracic cage to the upper lumbar spine creates an additional stress riser.

Inhibited anterior vertebral growth reflects the Heuter-Volkmann principle for vertebral apophyses and subsequent development of vertebral wedging. Neurologically such cases are liable to spinal compression because the spinal canal is abnormally narrowed by the short pedicles, as well as by compensatory hyperlordosis [16]. Pauli et al. suggested that a biophysical explanation of progression was secondary to a number of nearly uniform features in infants with achondroplasia, including hypotonia, macrocephaly, and generalized ligamentous laxity [17]. These features mean that when placed in a sitting position, a slumped, C-sitting posture arises, which can lead to anomalous gravitational forces causing remodeling of an intrinsically abnormal spine [18]. In this study, in 6 of 7 patients with TLK >20° at age 3, TLK progressed or remained at >20° at age 5, and in all except one TLK increased or remained at >20° at age 7. These results suggest that sagittal alignment at 3 years old determines future TLK in achondroplasia.

If mechanical stress is produced by uniform features, then prohibition of unsupported sitting and other strategies to decrease the time spent with gravity exerting disadvantageous forces should decrease the likelihood for kyphosis to progress. [19]. Pauli et al. found that counseling against unsupported sitting in the first 12–15 months of life was effective in markedly lowering the incidence of kyphosis progression in a longitudinal study [18]. Prohibition of unsupported sitting, good back support in other circumstances, avoidance of umbrella strollers and emphasis on time of prone positioning (“tummy time”) appear to be generally effective in reducing the risk that kyphosis will progress [14]. Another study recently confirmed the benefit of such behavioral strategies, as well as demonstrating that cases with more severe motor delays (presumably because of more severe hypotonia) are more likely to have persisting kyphotic curves [20]. This is similar to our data showing a significant difference in age at independent walking among subjects with and without TLK >20° at age 3.

With such interventions, nonetheless, about 30% of individuals will have a persistent TLK curve [20]. Pauli et al. developed treatment for those in whom more than a mild, fixed component of the kyphotic curve develops, using a modified thoracolumbosacral orthosis (TLSO) [17]. Similarly, in a retrospective evaluation, Xu et al. showed that bracing initiated early enough in life may be effective in reversing kyphotic curves that otherwise might subsequently require surgery [21]. In individuals whose parents were not counseled on preventive strategies, or in whom prevention and bracing fails, surgery is appropriate [6,7,22].

The limitations of this study include the small number of subjects. Inclusion of a larger number of patients could reveal a significant association between prognostic factors with progression. A longer follow-up until adult age is also needed to identify the natural history. However, this study is the first prospective long-term evaluation of progression in the sagittal plane in a cohort of nonsurgical patients with achondroplasia.

In summary, TLK is common in infants with achondroplasia. TLK >20° occurs in 76.2% of infants in the first 7 months of life. With acquisition of a sitting posture between 12 and 24 months, kyphosis is postulated to increase to 85.7%, but the rate decreases to 33.3% by age 5. In contrast to the decrease in TLK, lumbar lordosis and sacral slope increase with aging. In 6 of 7 patients with TLK >20° at age 3, TLK progressed or remained at >20° at age 5. All except one of these patients further progressed or still had TLK >20° at age 7. These results suggest that sagittal alignment at 3 years old determines future TLK in achondroplasia. Progression of deformity and neurological impairments require consideration in treatment planning.

## Conclusion

TLK is common in infants with achondroplasia. TLK >20° occurs in 76.2% of infants in the first 7 months of life. With acquisition of a sitting posture between 12 and 24 months, kyphosis is postulated to increase to 85.7%, but the rate decreases to 33.3% by age 5. In contrast to the decrease in TLK, lumbar lordosis and sacral slope increase with aging. In 6 of 7 patients with TLK >20° at age 3, TLK progressed or remained at >20° at age 5. All except one of these patients further progressed or still had TLK >20° at age 7. These results suggest that sagittal alignment at 3 years old determines future TLK in achondroplasia. Progression of deformity and neurological impairments require consideration in treatment planning.

## Declaration of Competing Interest

Japanese Ministry of Health, Labor, and Welfare Grants-in Aid for Scientific Research (C) (18K09025) and the General Insurance Association of Japan funds were received in support of this work.

## Appendix Supplementary materials

Image, application 1

Image, application 2
